# Stress, mental ill-health and functional somatic syndromes in incident and chronic sleep disturbance in a general adult population

**DOI:** 10.1080/21642850.2023.2184372

**Published:** 2023-03-06

**Authors:** Hampus Enkvist, Albin Öhman, Matias Pitkänen, Maria Nordin, Steven Nordin

**Affiliations:** Department of Psychology, Umeå University, Umeå, Sweden

**Keywords:** Anxiety, burnout, depression, insomnia, population-based

## Abstract

**Objective:**

Sleep disturbance may constitute health problems for the afflicted individual, but documentation of its chronicity is sparse. The objective was to investigate the extent to which incident and chronic sleep disturbance are associated with stress, mental ill-health and functional somatic syndromes.

**Design:**

This was a prospective, longitudinal study with 3-year interval between two assessments (T1 and T2), with a population-based sample forming groups with incident sleep disturbance (disturbance only at T2; *n* = 303), chronic sleep disturbance (disturbance at T1 and T2; *n* = 343) and without sleep disturbance (neither at T1 nor T2; *n* = 1421). Questionnaire data were used at T2 of physician-based diagnosis of anxiety disorder, depression, exhaustion syndrome, and functional somatic syndrome as well as of degree of stress, burnout, anxiety and depression.

**Results:**

Significant associations were found between chronic sleep disturbance and all four diagnoses (odds ratios = 1.74–2.19), whereas incident sleep disturbance was associated only with exhaustion syndrome and depression (odds ratios = 2.18–2.37). Degree of stress, burnout, anxiety and depression increased significantly from the referents to incident and chronic sleep disturbance, in that order (eta^2^ = 0.083–0.166), except for the two latter groups not differing in depression.

**Conclusion:**

The findings imply that healthcare professionals should be observant regarding various conditions of, apart from stress, mental ill-health and functional somatic syndromes in patients who present themselves with sleep disturbance, and in particular chronic disturbance.

## Introduction

Most of us have periods in life in which sleep is disturbed, since sleep patterns vary naturally over the months (Morin et al., 2014). However, periodic poor sleep turns into chronic sleep disturbance in 10–15% of the adult population, thus experiencing difficulties sleeping for more than three consecutive months, and about 30% of the adult population is estimated to suffer from occasional problems with disturbed sleep (Doghramji, 2006; Höglund et al., 2020). In one study as many as 56% of elderly women were found to suffer from sleep disturbance (Paparrigopoulos et al., 2010). Apart from female sex (Zhang & Wing, 2006), aging (Crowley, 2011; Ohayon et al., 2004), low education (Gellis et al., 2005; Talala et al., 2012), not living with a partner (Gosling et al., 2014), and lack of physical exercise (Kredlow et al., 2015) are known to be associated with sleep disturbance.

Both physical and mental health are affected by poor sleep. Results from the Penn State Adult Cohort revealed increased risk of heart disorder, ulcer, and allergy/asthma due to chronic poor sleep (Fernandez-Mendoza et al., 2012). Mental ill-health, foremost in the form of depression, has been shown to both affect and be affected by chronically disturbed sleep (Rothe et al., 2020; Singareddy et al., 2012; Vgontzas et al., 2012), but the risk of developing anxiety is also increased (Cox & Olatunji, 2020; Neckelmann et al., 2007).

Incident poor sleep is not as well investigated as chronically disturbed sleep, but Fernandez-Mendoza et al. (2012) showed that it too increases the risk of physical disorders, such as heart disorder and ulcer. Relapsing into disturbed sleep is mostly related to mental health, such as impaired mood, but somatic impairments do also elevate the likelihood of disturbed sleep (Ji et al., 2019).

Stress has with its increase in physiological and psychological activity been suggested to be the opposite of sleep (Åkerstedt, 2006), and stress is indeed related to poor sleep quality (van Laethem et al., 2015), with a reciprocal causal relationship (Nollet et al., 2020). Disturbed sleep may be a universal indicator of mental ill-health, and stress is likely to play a critical role in this context. Long-term stress with limited recovery often results in burnout (McEwen, 1998), which, in turn, is associated with disturbed sleep (Armon et al., 2008; Ekstedt et al., 2006; Grossi et al., 2003; Rothe et al., 2020; Söderström et al., 2012). Apart from stress and burnout, disturbed sleep has been shown to be associated with conditions such as anxiety, panic disorder, depression, and post-traumatic stress disorder (PTSD; Alvaro et al., 2013; Coles et al., 2015; Krystal & Thakur, 2008; Lee & Douglass, 2010; Reimann et al., 2001; Xu et al., 2012).

Functional somatic syndromes such as fibromyalgia, irritable bowel syndrome (IBS) and migraine are also known to be associated with burnout, anxiety and depression (Bernik et al., 2013; Epstein et al., 1999; Grassini & Nordin, 2017; Hausteiner-Wiehle & Henningsen, 2014; Kudielka et al., 2006). These syndromes refer to physical symptoms without an organic disease explanation, demonstrable structural changes or established biochemical abnormalities. Fibromyalgia can be described as widespread body pain in certain tender points as well as cognitive symptoms, fatigue, and a number of somatic symptoms (Wolfe et al., 2010). IBS is associated with abdominal pain or discomfort associated with defecation or a change in bowel habit and with features of disordered defecation (Longstreth et al., 2006), and migraine with disabling headache attacks, typically unilateral with a pulsating quality. These pain syndromes are also associated with disturbed sleep (Bellini et al., 2011; Hamilton et al., 2008; Headache Classification Committee of the International Headache Society, 2013; Roizenblatt et al., 2011). In fact, there is most likely to be a bidirectional relationship between pain and sleep, in which pain causes difficulties initiating sleep, and sleep disturbance exaggerates the pain (Arnison et al., 2022). Individuals who exhibit both somatic syndromes and disturbed sleep have more complaints about mental ill-health such as stress, depression and anxiety (Ionescu et al., 2021).

It is reasonable to argue that professionals in primary healthcare should be particularly observant regarding conditions of mental ill-health and functional somatic syndromes in patients who show signs of chronic sleep disturbance. Apart from comparing chronic sleep disturbance with stable good sleep over time, it is informative to compare these conditions with incident sleep disturbance since this is a common condition that may affect health in a different way than chronic sleep disturbance (Yang et al., 2013).

The aim of the present study was to better understand comorbidity in sleep disturbance with stress, mental ill-health and functional somatic syndromes, depending on the duration of the sleep disturbance. Longitudinal data were used at a first (T1) and second (T2) assessment, separated by three years. The objective was to test the hypotheses of incident and chronic sleep disturbance at T2 being associated with (i) self-reports of diagnoses given by a physician of exhaustion syndrome (‘clinical burnout’), anxiety disorder, depression and functional somatic syndromes, and (ii) high level of stress, burnout, anxiety and depression in a general adult population, and that the level of stress, burnout, anxiety and depression would be stronger in chronic than in incident sleep disturbance. Incident sleep disturbance (disturbance at T2, but not T1) and chronic sleep disturbance (at both T1 and T2) were compared with a reference group defined as not having disturbed sleep at either T1 or T2. An anxiety disorder included the diagnoses generalized anxiety disorder (GAD), panic disorder and/or (PTSD), and functional somatic syndromes included fibromyalgia, IBS and/or migraine.

## Materials and methods

### Study population and sample

Data were used from the Västerbotten Environmental Health Study, which investigates mental and somatic health in a general Swedish adult population (Palmquist et al., 2014). The county of Västerbotten in Northern Sweden has a population of approximately 270,000 inhabitants (about 200,000 aged 18–79 years) and an age and sex distribution that is very similar to the general Swedish population (Statistics Sweden, 2010).

A random sample of 8520 individuals aged 18–79 years from Västerbotten was by means of the municipal register invited to participate at T1 in 2010. The sample was stratified for age and sex according to the age strata 18–29, 30–39, 40–49, 50–59, 60–69 and 70–79 years. Among the invited, 3406 (40.0%) agreed to participate. Of these, 3181 were still alive and living in Västerbotten at T2 in 2013, of which 2336 (73.4%) agreed to participate again. Numbers of respondents across age and sex strata at T1 and T2 are given in Table 1.

**Table 1.** Numbers of respondents (and response rate in percentage) across age and sex strata at a first (T1) and second (T2) assessment.

T1			T2	
Age (years)	Women	Men	Women	Men
18–29	307 (32.1)	179 (17.3)	137 (59.5)	76 (54.7)
30–39	266 (40.3)	177 (24.7)	165 (66.3)	90 (53.9)
40–49	288 (40.5)	230 (31.0)	197 (71.9)	139 (61.8)
50–59	367 (50.9)	295 (39.5)	283 (79.3)	226 (77.4)
60–69	405 (58.4)	356 (50.7)	324 (82.2)	293 (84.2)
70–79	265 (53.8)	271 (63.9)	200 (50.8)	206 (80.8)
Total sample	1898 (45.2)	1508 (34.9)	1306 (74.5)	1030 (72.2)

### Single questions

Data at T2 from single questions and questionnaire instruments were used to study factors associated with sleep disturbance. Apart from single questions used for a background description of the participants, the question ‘Have you been diagnosed with this condition by a physician?’ was used to assess self-report of having been given a diagnosis of GAD, panic disorder, PTSD, depression, exhaustion syndrome, fibromyalgia, IBS and migraine, which were on a list from which to be chosen. Exhaustion syndrome is a diagnosis used in Sweden for cases with similar symptoms of ‘clinical burnout.’ It is defined as distinct mental and somatic impact after long-term stress, with diagnostic criteria that include physical and psychological exhaustion, lack of mental energy, a certain symptom pattern, symptoms causing clinical suffering or reduced capacity at work or in social life, and the condition not being caused by substances or somatic disease (Swedish National Board of Health and Welfare, 2003).

### Questionnaire instruments

Perceived, rather than objectively measured sleep, was assessed since perceived poor sleep may be more relevant in this context as it may lead to worry and stress, which, in turn, may perpetuate sleep disturbance and potentially lead to disease (Roth & Roehrs, 2003). The Karolinska Sleep Questionnaire (KSQ; Åkerstedt et al., 2008; Nordin et al., 2013) was used for this purposes. The KSQ consists of 18 items and assesses the dimensions poor sleep quality, non-restorative sleep, daytime sleepiness, and sleep apnea. The response options for the KSQ are (0) Never, (1) Seldom (occasionally), (2) Sometimes (several times per month), (3) Often (1–2 times per week), (4) Most of the time (3–4 times per week), and (5) Always (5 times or more per week), and the participants are asked to consider their sleep for the past three months. The score for each subscale is calculated as the mean across items. The condition of sleep disturbance was based on an index of the two dimensions sleep quality and non-restorative sleep of the KSQ. The items in the sleep quality dimension were ‘difficulties falling asleep,’ ‘repeated awakenings with difficulties falling asleep again,’ ‘premature awakenings, and disturbed/restless sleep,’ and those in the non-restorative sleep dimension were ‘difficulties waking up,’ ‘not well-rested on awakening,’ and ‘feelings of being exhausted at awakening.’ The inclusion criterion for sleep disturbance required one or more of the seven items (symptoms) to be rated as 4 or 5 (≥3 times per week). This is in accordance with the DSM-5 criteria for insomnia, which includes sleep difficulty occurring at least three nights per week for at past 3 months. Based on this criterion, incident disturbed sleep was defined as disturbed sleep at T2, but not at T1, and chronic disturbed sleep as disturbed sleep at both T1 and T2. Referents were defined as not having disturbed sleep either at T1 or T2. The KSQ has good reliability, construct validity, and criterion validity (Nordin et al., 2013). Cronbach’s alpha for the present sample was 0.84 for the items of the sleep disturbance index.

The Swedish version (Nordin & Nordin, 2013) of the 10-item Perceived Stress Scale (PSS-10; Cohen & Williamson, 1988) was used to measure degree to which situations in the person’s life are appraised as stressful, with items that tap how unpredictable, uncontrollable, and overloaded respondents have found their lives in the last month (e.g. ‘How often have you felt that you were unable to control the important things in your life?’). The scale ranges from 0 to 40 (high score representing high stress level). It has documented good reliability and construct validity (Cohen & Williamson, 1988; Nordin & Nordin, 2013). Cronbach’s alpha for the present sample was 0.83 for the PSS-10.

The Shirom Melamed Burnout Questionnaire (SMBQ; Melamed et al., 1992), translated to Swedish (Grossi et al., 2003), was used to assess burnout in terms of physical fatigue, cognitive weariness, tension and listlessness. The global scale (ranging from 1 to 7 in score with high score indicating high level of burnout) was used for the present purposes which is based on all 22 items. The SMBQ has good construct validity and reliability (Grossi et al., 2003). Cronbach’s alpha for the present sample was 0.95 for the SMBQ.

The Swedish version (Lisspers et al., 1997) of the Hospital Anxiety and Depression Scale (HADS; Zigmond & Snaith, 1983) was used to assess anxiety (HADS-A) and depression (HADS-D). It consists of seven items for anxiety (e.g. ‘Worrying thoughts go through my mind’) and seven for depression (e.g. ‘I have lost interest in my appearance’) regarding the past week. Each scale ranges from 0 to 21 (high score representing high anxiety and depression). The HADS has good discriminant and concurrent validity and good internal consistency (Bjelland et al., 2002). Cronbach’s alpha for the present sample was 0.85 for the HADS-A and 0.84 for the HADS-D.

### Procedure

The participants were mailed the questionnaire, to be returned by mail with prepaid postage. It was sent together with a letter containing information regarding confidentiality, intended use of the data and voluntariness. A reminder was sent to non-responders after fully three weeks, and, if needed, an additional reminder and a new copy of the questionnaire after another three weeks. The participants responded to the questionnaire during the period March to April at T1 (2010) and T2 (2013). The data collection was conducted in accordance with the Helsinki Declaration, and is approved by the Umeå Regional Ethics Board (Dnr 09-171M) and the Swedish Ethical Review Authority (Dnr 2022-05265-02). All participants gave their informed consent to participate.

### Statistical analysis

Missing values were estimated with multiple imputation using fully conditional Markov chain Monte Carlo procedure (Schunk, 2008) with 10 maximum iterations by means of which five imputations were generated, and the obtained estimate value averaged. The percentage of the missing data from the questionnaire instruments at T2 was 1.49% for the PSS-10, 2.47% for the SMBQ, and 1.24% for the HADS. Corresponding percentage for the KSQ was 2.43% at T1 and 2.80% at T2.

Based on the sleep disturbance index, an incident sleep disturbance group, a chronic sleep disturbance group, and a reference group were formed and compared on demographics, physical exercise and sleep variables at T2 with overall one-way analysis of variance (ANOVA) and chi-square analysis, followed by post-hoc analysis with ANOVA and chi-square analysis.

Percentages of those with sleep disturbance who had been given a diagnosis of anxiety disorder (GAD, panic disorder and/or PTSD), depression, exhaustion syndrome, and functional somatic syndrome (fibromyalgia, IBS and/or migraine) were calculated. As a further step, binary logistic regression analyses were conducted to study these diagnoses as being associated with incident and chronic sleep disturbance, providing odds ratios and 95% confidence intervals. Age, sex, being married or living with partner and physical exercise at T2 were controlled for since these variables differed significantly between groups. Score on sleep apnea (disruptive breathing), assessed with the Sleep apnea subscale of the KSQ (Nordin et al., 2013) was also controlled for.

Group differences in degree of stress, burnout, anxiety and depression were tested statistically with overall one-way analysis of covariance (ANCOVA), with age, sex, being married or living with partner, physical exercise and sleep apnea as covariates, followed by post-hoc ANCOVAs. Partial eta^2^ was used as a measure of effect size. The α-level was set at 0.05. SPSS Statistics 26 (IBM Corporation, New York) was used for the statistical analyses.

### Ethics statement

The data collection was conducted in accordance with the Helsinki Declaration, and is approved by the Umeå Regional Ethics Board (Dnr 09-171M) and the Swedish Ethical Review Authority (Dnr 2022-05265-02). All participants gave their informed consent to participate.

## Results

Among the 2336 participants, 303 (13.0%) met the criterion for incident sleep disturbance, 343 (14.7%) for chronic sleep disturbance, and 1421 (60.8%) for being a referent. Remaining 269 (11.5%) were excluded from further analysis. The case and referent groups are described on demographics, physical exercise and sleep variables in Table 2. Group differences were found for age, sex, being married or living with partner, physical exercise, time slept, sleep duration needed, extent of sufficient sleep, and scores on the four KSQ subscales, but not for education. The Pearson correlation coefficients for the six pairwise correlations between scores on the PSS-10, SMBQ, HADS-A and HADS-D ranged from 0.55 to 0.70 at T1 and from 0.53 to 0.70 (*p* < 0.001 in all cases) at T2.

**Table 2.** Description of the groups at a second assessment (T2), showing results from post-hoc analysis of variance and chi-square analysis.

	Referents	Incident	Chronic	Post-hoc
	(R; *n *= 1421)	sleep	sleep	
		disturbance	disturbance	
		(I; *n *= 303)	(C; *n *= 343)	
Age, mean±SD years	58.2±14.7	54.8±17.2	54.6±16.1	R>I=C
Women, *n* (%)	728 (51.2)	179 (59.1)	232 (67.6)	R<I=C
University education	590 (41.9)	116 (38.8)	138 (40.1)	
Married or living with partner, *n* (%)	908 (64.4)	167 (55.9)	196 (57.8)	R>I & C, I=C
Physical exercise ≥twice/week, *n* (%)	1009 (71.6)	195 (64.8)	228 (67.5)	R>I, R=C, I=C
Time slept/night, mean±SD hours	7.14±0.95	6.76±1.29	6.43±1.55	R>I>C
Sleep duration needed/night,				
mean±SD hours	7.47±0.83	7.71±1.20	7.92±1.55	R<I=C
Extent of sufficient sleep, n (%)				R>I>C
Yes, enough at large or definitely				
Enough	1125 (80.0)	140 (47.9)	80 (24.0)	
No, somewhat not enough	251 (17.9)	94 (32.2)	129 (38.7)	
No, far from enough or clearly				
not enough	27 (2.0)	58 (19.9)	125 (37.5)	
KSQ, mean±SD				
Poor sleep quality	1.18±0.63	2.40±1.10	2.73±1.02	R<I<C
Non-restorative sleep	0.92±0.60	2.28±1.02	2.46±1.09	R<I=C
Daytime sleepiness	0.85±0.60	1.76±1.08	1.87±1.02	R<I=C
Sleep apnea	0.61±0.77	1.15±1.32	1.07±1.25	R<I=C

KSQ = Karolinska Sleep Questionnaire.

**Figure 1.**
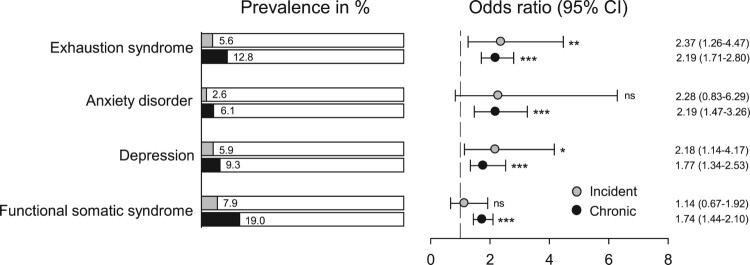
Prevalence of self-report of physician-based diagnoses in incident and chronic sleep disturbance. Odds ratios, confidence intervals (CIs) and *p*-values (*p < 0.05, **p < 0.01, ***p < 0.001, ^ns^non-significant) for comorbidity with these diagnoses are given when adjusted for age, sex, married or living with partner, physical exercise, and sleep apnea. Referents were used as a reference group. The vertical dashed line represents an odds ratio of unity.

Percentages of the participants in the incident and chronic sleep disturbance groups who at T2 reported having been given a diagnosis of anxiety disorder, depression, exhaustion syndrome, and functional somatic syndrome by a physician are shown in Figure 1. The prevalence rates ranged between 2.6% and 7.9% in the incident group, and between 6.1% and 19.0% in the chronic group. The figure also shows ORs for these diagnoses, which were significantly larger than unity for all four diagnoses in the chronic group (1.74–2.19), and for exhaustion syndrome and depression in the incident group (2.18–2.37).

**Figure 2.**
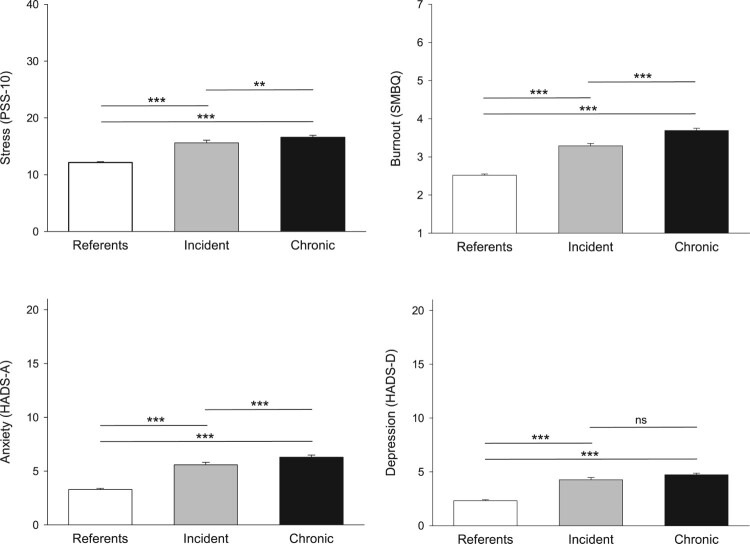
Mean ± SE scores on measures of stress and mental ill-health in the incident and chronic sleep disturbance and referent groups, and results from post-hoc comparisons (**p < 0.01, ***p < 0.001, ^ns^non-significant).

Mean scores at T2 on stress, burnout, anxiety and depression for the three sleep groups are presented in Figure 2. Results on the overall ANCOVAs showed significant group differences for stress (*F* = 92.42, partial eta^2^ = 0.083), burnout (*F* = 201.92, partial eta^2^ = 0.166), anxiety (*F* = 131.62, partial eta^2^ = 0.115) and depression (*F* = 121.44, partial eta^2^ = 0.107) for the three groups. Post-hoc ANCOVAs showed that the degree of all four conditions increased significantly from the referents to the incident and chronic sleep disturbance groups, in that order, expect for the two latter groups that did not differ in depression (Figure 2).

## Discussion

The present objective was to test hypotheses of associations in incident and chronic sleep disturbance with self-reports of physician-based diagnosis of psychiatric conditions of mental ill-health and functional somatic syndromes, and with level of stress and different types of mental ill-health in a general population. The results provide support for the hypotheses regarding chronic sleep disturbance. Thus, diagnoses of anxiety disorder, depression, exhaustion syndrome and functional somatic syndrome had ORs that were significantly larger than unity, with a 1.74- to 2.19-fold increased risk. In absolute terms the prevalence rates for these diagnoses in chronic sleep disturbance ranged from 6.1% to 19.0%. Furthermore, compared to the reference group, the chronic sleep disturbance group showed higher level of stress, burnout, anxiety and depression. The effect sizes (partial eta^2^ ranging from 0.083 to 0.166) from the overall ANCOVAs can be considered as medium to large (Cohen, 1988). The results on degree of mental ill-health for burnout, anxiety and depression can be considered as supplementary to those on diagnoses of anxiety disorder, depression and exhaustion syndrome, and together the findings indeed suggest that anxiety/anxiety disorder, depression and burnout/exhaustion syndrome are associated with chronic sleep disturbance.

The present findings corroborate previous findings in that stress, depression, anxiety and somatic symptoms are associated with chronic sleep (Fernandez-Mendoza et al., 2012; Ionescu et al., 2021; Neckelmann et al., 2007; Singareddy et al., 2012; Vgontzas et al., 2012).

In line with Fernandez-Mendoza et al. (2012), incident sleep disturbance indicates an increased risk of mental and somatic symptoms. Our results provide partial support for the hypotheses regarding incident sleep disturbance. Whereas the ORs were larger than unity for all four diagnoses, the difference was significant for exhaustion syndrome and depression, but not for anxiety disorder or functional somatic syndrome. The prevalence rates for these diagnoses in this group ranged from 2.6% to 7.9%. As for chronic sleep difficulty, the group with incident sleep difficulty showed higher level than the reference group on stress, burnout, anxiety and depression.

As would be expected, there was a general trend in degree of mental distress being higher in chronic than in incident sleep disturbance. The degree differed significantly between the two sleep disturbance groups for stress, burnout and anxiety, whereas it did not reach significance for depression. However, a consistent trend of this kind was not found for the ORs, which may partly be due to the use of categorical data typically being less sensitive than use of continuous data.

Several mechanisms may contribute to sleep disturbance being associated with the studied health conditions. Less than normal deep sleep has been demonstrated in anxiety (Lee & Douglass, 2010), that may be due to a change in the circadian rhythm (Coles et al., 2015). Stress-induced elevation of inflammatory markers (interleukin-6 and serum intercellular adhesion molecule) has been suggested to cause sleep disturbance in depression (Motivala et al., 2005). Panic attacks at night resulting in being afraid to fall asleep may explain disturbed sleep in panic disorder, and fragmented sleep and increased motor activity and arousal in PTSD (Lee & Douglass, 2010). Fragmented sleep and increased arousal has been found also in burnout as well as more stage-1 sleep, less slow-wave sleep and rapid eye movement sleep, and a lower delta power density in non-rapid eye movement sleep (Ekstedt et al., 2006). There is support for pain being a strong cause of disturbed sleep (Hamilton et al., 2008; Osorio et al., 2006; Roizenblatt et al., 2011), which may explain disturbed sleep in fibromyalgia, migraine and probably also IBS. Kellow et al. (1990) reported episodes of clustered contractions in IBS that were associated with incident abdominal pain and discomfort.

Level of stress was found to be associated with sleep disturbance, and is likely to play a crucial role in the relation between mental ill-health/functional somatic syndromes and disturbed sleep. Åkerstedt (2006) describes the stress – sleep interaction as activation of the sympatho-adreno-medullary and hypothalamo-pituitary-adrenocortical systems, with increased levels of catecholamines, cortisol, adrenocorticotropic and corticotropin releasing hormones as well as increased activation of the cardiovascular system. He further argues that stress systems interact with the endocrine, gastrointestinal, and immune systems through complex stimulatory and inhibitory feedback pathways.

Comorbidity between anxiety, depression and burnout is well documented (Kaufman & Charney, 2000; Swedish National Board of Health and Welfare, 2003). Regarding functional somatic syndromes, fibromyalgia has shown comorbidity with anxiety, panic disorder, PTSD and depression (Bernik et al., 2013; Hudson et al., 1992; Toussaint et al., 2017), IBS with GAD, panic disorder, PTSD and depression (Ståhlberg et al., 2018), and migraine with anxiety, panic disorder, exhaustion syndrome and depression (Grassini & Nordin, 2017). This implies that several of the described mechanisms that can contribute to disturbed sleep may be at hand in the studied conditions. This is supported by the rather high correlation coefficients (0.53–0.70) between scores on the PSS-10, SMBQ, HADS-A and HADS-D.

Although outside the scope of the present study, there is support for a bidirectional relation between sleep disturbance and both anxiety (Alvaro et al., 2013), depression (Alvaro et al., 2013; Reimann et al., 2001; Xu et al., 2012) and stress/burnout/exhaustion syndrome (Armon et al., 2008; Söderström et al., 2012). Ohayon and Roth (2003) reported that in most cases of mood disorders, sleep disturbance appear before or at the same time as the mood disorder, whereas sleep disturbance appears mostly at the same time or after an anxiety disorder. Apart from one condition leading to the development of a new condition, a certain condition may also aggravate and sustain an existing condition. For example, sleep disturbance has been suggested to hamper recovery from burnout and exhaustion syndrome (Grossi et al., 2015; Sonnenschein et al., 2007). There may also be a bidirectional relationship between sleep disturbance and functional somatic syndromes, predominantly due to the pain in these syndromes causing difficulties initiating sleep, and sleep disturbance exaggerating the pain (Arnison et al., 2022). Although longitudinal data were used in the present study, the focus was on comparing incident and chronic disturbed sleep in their associations with other health issues at T2, using T1 data only to determine whether the sleep disturbance at T2 was considered chronic of not. Future studies may use designs that in a more direct sense enables investigation of causal direction regarding sleep disturbance and stress/mental ill-health/functional somatic syndromes.

The present results suggest that duration of the sleep disturbance plays a role for its association with stress, mental ill-health and functional somatic syndromes, although associations were found also in incident sleep disturbance. The generally stronger associations with mental ill-health and functional somatic syndromes in chronic compared to incident sleep disturbance is likely to be due, at least partly, to poorer sleep quality in chronic sleep disturbance (Table 2). A clinical implication of these findings is that in cases of signs of sleep disturbance healthcare professionals should not only investigate current sleep disturbance, but inquire information also about the patient’s history of sleep disturbance. Another implication is that healthcare professionals should investigate potential stress, mental ill-health and functional somatic syndromes in cases of sleep disturbance, and investigate potential sleep disturbance in cases of stress, mental ill-health and functional somatic syndromes.

Strengths of the present study include use of a population-based sample, a large sample size, and that the county of Västerbotten has an age and sex distribution that is similar to that of Sweden in general (Statistics Sweden, 2010). Retrospective information about the participants’ sleep behavior three years earlier based on their memory is highly questionable regarding its reliability. For this reason, the longitudinal design enabling actual data collection on sleep disturbance also at T1, three year prior to the main assessment at T2, is another strength of this study.

The study did also have limitations. Whereas the response rate at T2 (73.4%) can be considered as satisfactory, it was rather low at T1 (40.0%). Notably though, effects of low response rate have been shown to vary little between response rates of 30–70% (Galea & Tracy, 2007). A potential selection bias may therefore be more of a concern. It is possible that persons with severe mental ill-health, in particular depression, were less likely to participate in the study. If so, the variance in the data would be smaller than otherwise, and if so, the obtained effect sizes would be underestimated. Another limitation is the absence of information about the time of the diagnoses given by a physician, which restricts us to refer to these as lifetime diagnoses. However, it is of interest to note that assessment six months after initial assessment in a non-patient population has demonstrated high reliability of lifetime assessment of depression (Andreasen et al., 1981). Furthermore, chronic sleep disturbance was defined here as disturbed sleep for a 3-month period both at baseline and 3-year follow-up. This calls for caution since information about sleep disturbance between the two measurements is not available. However, it is more likely to rate one’s sleep as disturbed if it was disturbed at a prior occasion, indicating a vicious circle once sleep has become disturbed (Morin et al., 2009). Yet another limitation is the large uncertainty of the size of the ORs for the diagnoses in incident sleep disturbance due to large CIs. A final limitation regards the prevalence rates for incident (13.0%) and chronic (14.7%) sleep disturbance in 2013. Whereas associations between sleep disturbance, on the one hand, and stress, mental ill-health and functional somatic syndromes, on the other hand, may change only marginally over time, the prevalence of sleep disturbance may have changed since the assessment in 2013. However, prevalence of sleep disturbance was not the main focus of the present study.

In conclusion, despite the limitations, the present results suggest an increased risk of a diagnosis of exhaustion syndrome and depression in incident and chronic sleep disturbance, and an increased risk of a diagnosis of anxiety disorder and functional somatic syndrome in the chronic condition. The results further suggest increased level of stress, burnout, anxiety and depression in incident and chronic sleep disturbance. From a clinical perspective, these findings imply that health care professionals, apart from stress, should be observant regarding various conditions of mental ill-health and functional somatic syndromes in patients who present with sleep disturbance, in particular with chronic disturbance.

## Data Availability

Data used for this analysis will be made available upon request.
